# Implications of Decreased Expression of miR-125a with Respect to Its Variant Allele in the Pathogenesis of Recurrent Pregnancy Loss: A Study in a High Incidence Zone

**DOI:** 10.3390/jcm11133834

**Published:** 2022-07-01

**Authors:** Usma Manzoor, Arshad A. Pandith, Ina Amin, Saima Wani, Dheera Sanadhya, Tawseef A. Lone, Hyder Mir, Bilal Ahamad Paray, Aneela Gulnaz, Iqra Anwar, Abida Ahmad, Qurat Ul Aein

**Affiliations:** 1 Advanced Centre for Human Genetics, Sher-I-Kashmir Institute of Medical Sciences (SKIMS), Srinagar 190011, India; usmamanzoor21@gmail.com (U.M.); inaamin@gmal.com (I.A.); iqraanwar@gmal.com (I.A.); quratt4@gmail.com (Q.U.A.); 2 School of Life and Basic Sciences, Jaipur National University, Jaipur 302017, India; dheerasanadhya2@gmal.com; 3 Department of Clinical Biochemistry, University of Kashmir, Srinagar 190006, India; 4 Department of Obstetrics and Gynecology, Sher-I-Kashmir Institute of Medical Sciences (SKIMS), Srinagar 190011, India; ark.saima@yahoo.com.com (S.W.); dr_abida@rediffmail.com (A.A.); 5 Department of General Surgery, Sher-I-Kashmir Institute of Medical Sciences (SKIMS), Srinagar 190011, India; tawseeflone34@gmail.com; 6 Influenza Lab, Internal and Pulmonary Medicine, Sher-I-Kashmir Institute of Medical Sciences (SKIMS), Srinagar 190011, India; mirhyder8@gmail.com; 7 Department of Zoology, College of Science, King Saud University, P.O. Box 2455, Riyadh 11451, Saudi Arabia; bparay@ksu.edu.sa; 8 College of Pharmacy, Woosuk University, Wanju-gun 55338, Korea; draneela@woosuk.ac.kr

**Keywords:** recurrent pregnancy loss, product of conception, single nucleotide polymorphism, expression, sequencing

## Abstract

Pregnancy is controlled by several types of genes and the regulation of their expression is tightly controlled by miRNAs. The present study was carried out to explore the association between miR-125a polymorphic sequence variation and its expression and recurrent pregnancy loss (RPL) compared to full-term healthy controls. A total of 150 women that had experienced two or more RPLs and 180 healthy controls (two or more full-term pregnancies) were recruited, along with 50 product of conception (POC) samples from the corresponding RPL patients, and evaluated for miR-125a SNPs by the polymerase chain reaction-restriction fragment length polymorphism method (PCR-RFLP), which was confirmed by high resolution melting (HRM)/DNA sequencing. Additionally, the expression of miR-125a was quantified with q–PCR in the maternal plasma of 40 corresponding RPL patients against healthy controls. The frequency of variant genotype CC was significantly higher in RPL cases (19.3%) than controls (10.5%), with an odds ratio of >2 (*p* = 0.025). The expression levels of miR-125a were markedly decreased in RPL cases compared to healthy controls (*p* < 0.05). Variant genotype CC was found significantly more often in RPL cases than controls (0.34 vs. 0.20; *p* < 0.05).In this study, miR-125a rs12976445 C/T revealed that the homozygous CC genotype and C allele were associated with the risk of RPL and significant expression indicates that miR-125a has an important role in RPL etiopathogenesis.

## 1. Introduction

Recurrent pregnancy loss (RPL) provides a fundamental insight into the processes of embryogenesis and implantation. Epidemiological research has showed that the disorder might have a complex etiology with a possible hereditary susceptibility and environmental influences [1]. Several studies have reported the frequency of RPL to be between 0.5% and 2.3%, as described by Sugiura-Ogasawara et al., 2013 in the Japanese population [2,3]. In our previous study, it was observed that the frequency of RPL is very high in the Kashmiri region (North India), and interestingly, a high number of cases were observed in consanguineous groups with a known family history. Apart from consanguinity and family history, the patterns of RPL events that were observed defy the norm of it being more prevalent in advanced maternal age. Indeed, RPL was observed with almost equal frequency in both age groups (<30 and >30 years) [4]. It has been observed that both parental and embryo/fetal factors are associated with RPL. Parental factors include parental chromosomal translocations, maternal thyroid or prolactin disease or diabetes mellitus, endometrial changes, intrauterine abnormalities, and antiphospholipid antibodies. A recent study also found evidence showing an association between inherited thrombophilia and recurrent early pregnancy failure. On the basis of earlier reports, hematological factors such as factor V Leiden, activated protein C resistance, fasting homocysteine, antiphospholipid antibodies, and the prothrombin gene can also be taken into consideration in cases of RPL [5].

Recently, the focus of many studies has switched from mRNAs to non-coding RNAs (ncRNAs) as a primary regulator of the human genome. In ncRNAs, a certain type of small RNA molecules known as micro RNAs (miRNAs) are found in every eukaryotic cell because these may specifically attach to the mRNAs, which leads to translational repression and gene silencing [6]. The biosynthesis of miRNAs primarily involves two pathways: the canonical pathway and non-canonical pathway. In the canonical pathway, initially, the RNA polymerase II transcripts the long primary miRNA (pri-miRNA) transcript [7] and the resulting pri- miRNA is processed by the Drosha nuclear enzyme RNase III and the DiGeorge Essential Region 8 (DGCR8), which then produces pre-miRNA of ±70 nt in length [8]. The nuclear export allows nuclease Dicer cleavage, resulting in a duplex of ~22 nt miRNA/miRNA [9]. Genetic changes that affect miRNA at a physiological level or target specificity may have serious effects on cellular protein levels and thus contribute to various diseases [10]. Many genomic profiling studies have found that miRNAs exhibit decreased expression in a variety of malignancies, including pancreatic cancer, breast cancer, prostate cancer, liver cancer, colon cancer, and ovarian cancer, implying that miRNAs negatively regulate cancer progression [11,12,13,14,15,16,17,18,19]. As per the recent review conducted by Barchitta et al. on the identification of miRNA profiles in RPL cases, the findings showed multiple miRNAs that exhibited low expression in cases of unexplained spontaneous pregnancy loss compared to controls [20]. Polymorphic sequence variations in miRNA genes could possibly remodel various biological processes by affecting the processing, and finally, the target selection of miRNAs. Moreover, collective evidence from neoteric reports have indicated that multiple miRNAs are associated with one or more facets of pregnancy and its outcomes [21]. A recent study reported that sufficient levels of maternal plasma miRNAs, particularly miR-21, miR-126 and miR-182 are important for pregnancy and could serve as possible targets for diagnosis and intervention in RPL cases [22]. Interestingly, the concentration and profile of miRNAs in plasma may be utilized for the detection of adverse pregnancy outcomes as they seems to cross the placenta and appear in the maternal circulation [23].

In human chromosomes, miR-125a is mapped to chromosome 19q13.41 and plays a critical role in organ growth and the development of adult tissues [24,25]. Studies have examined and described the effect of a pri-miRNA single nucleotide polymorphism (SNP) rs12975333 (G > T) on processing for miR-125a. The expression and activity of the miRNA are affected miR-125a rs12975333-T, a minor variant, reduces the binding of pri-miR-125a to the DGCR8, which is a major component in the biogenesis of miRNA, leading to incorrect pri- to pre-miR processing and low levels of mature miR-125a [26]. It has also been reported that two variant alleles in rs12976445 and rs41275794 polymorphism led to the defective production of miR-125a, which is associated with an elevated risk for RPL [10]. In a Chinese population, Hu et al., 2011 discovered that pre-rs12976445 polymorphism resulted in a significant reduction in the amount of mature miR-125a by affecting mature miRNA processing, resulting in less effective regulation of the leukemia inhibitory factor receptor (LIFR)gene and an increased risk of recurrent pregnancy loss [27]. Furthermore, it has been suggested that the A > G mutation in miR-125a SNPs can affect the expression of the erythroblastic leukemia viral oncogene homolog 2 (ERBB2) gene by changing the production of miR-125a [28]. These findings collectively suggest the important role of these two genes in stroma and trophoblast growth and differentiation during early pregnancy in humans. In a recent review, it was observed that in the miR-125a A-T haplotype, the expression of the mature miR-125a was decreased, leading to an increase in LIFR and ERBB2 expression. The LIFR ectopic expression will partially block the activities of LIF and disrupt the post-implantation development of trophoblasts and their differentiation. Excessive VEGF synthesis in stromas and trophoblasts can promote the ectopic expression of ERBB2 and result in an undue invasion of trophoblast cells. This is a plausible reason as to why miR-125a T-A may have a prominent role in RPL susceptibility. Furthermore, it has also been observed in a previous study that consanguineous marriages continue to be a critical predictor of adverse pregnancy outcomes in India [29]. Thus, keeping in mind our highly in-bred population with a huge burden of recurrent miscarriages [4], the present study was designed to investigate the role of miR-125a SNPs and its expression in RPL in patients in Kashmir (North India).

## 2. Materials and Methods

### 2.1. Study Population

The current study was conducted at the Advanced Centre for Human Genetics at the Sheri-I-Kashmir Institute of Medical Sciences (SKIMS), Srinagar, India, between 2017 and 2020. The study was approved by the Regional Ethics Committee of SKIMS (IEC-SKIMS). The study included 200 women who had experienced at least two recurrent pregnancy losses at ≤20 weeks of gestation, who were recruited from the Department of Gynecology and Obstetrics, SKIMS. Serial ultrasound, parental Karyograms (done by Karyotyping through the GTG banding technique), hormonal profiles, sugar levels, toxoplasmosis, rubella, cytomegalovirus, herpes simplex (TORCH) testing, HIV testing, anti-phospholipid antibodies, and lupus anticoagulant were all used to rule out additional reasons for abortion. In addition, among 200 RPL patients, 50 samples of product of conception (POC) were obtained in normal saline (50–100 mg from each patient) and kept at −20 °C until DNA extraction. Two hundred and forty women matched for age and ethnicity, with at least two live births and without a previous history of abortion or pregnancy-associated complications served as the control group, and were also recruited from the Department of Gynecology and Obstetrics, SKIMS. A path map showing the selection of cases with RPL and the control group is presented in the Supplementary Materials, Figure S1.

### 2.2. Sample Collection

A total of 5 mL of peripheral blood was collected in EDTA vials from patients with RPL and healthy controls. Immediately, plasma was collected from 3 mL of the sample and kept at −80 °C until further processing for miRNA extraction and the remaining blood sample was stored at −80 °C until it was processed for DNA extraction. POC samples (n = 50) were obtained in normal saline (50–100 mg from each patient) and kept at −20 °C until they were processed for DNA extraction. After obtaining written pre-informed consent from all participants, clinical information, such as age, number of miscarriages, and family history of RPL, etc., was recorded for each patient. Using the nMaster2.0 statistical program, the sample size was determined as >80%. All demographic information about the patients and controls is provided in the Supplementary Materials, Table S1.

### 2.3. DNA Extraction

DNA was extracted from POC tissue and/or blood samples for genetic analysis using a DNA extraction kit (Qiagen, Hidlen, Germany), or the conventional phenol chloroform/isoamyl technique. Spectrophotometer absorbance at 260 nm and 280 nm was used to assess the quality and quantity of the extracted DNA (Eppendorf AG; Serial No: 6137EQ102539; Hamburg, Germany). The extracted DNA was kept at −20 °C until miR-125a SNPs were analyzed.

### 2.4. miRNA Extraction from Plasma

To harvest cell-free plasma, 3 mL of whole blood samples were centrifuged twice at 1200 g for 10 min at room temperature and the supernatant plasma was quickly removed and stored at −80 °C until further processing. The miRNeasy Plasma Kit (Qiagen, Hilden, Germany) was used to extract miRNAs from plasma samples. The Quantus™ Fluorometer, in combination with the Invitrogen microRNA Assay Kit (E5150-Quantus Fluorometer Promega, Madison, Wisconsin, USA), was used to measure low levels of miRNA in the plasma. The assay quantifies miRNA in solution at a final assay concentration of 0.05–100 ng/µL. Additionally, the confirmation of successful miRNA extraction was indicated by a bright 5S band by gel electrophoresis that signified successful extraction of miRNA.

### 2.5. Genotyping of Polymorphisms by PCR-RFLP

PCR-RFLP analysis was employed in 150 patient samples from a total of 200 patients to differentiate different genotypes of the miR-125ars12976445 C/T and rs10404453 A/G polymorphic sequence variants. Patients with factors that are likely to cause pregnancy loss were excluded from the genotype analysis. Only patients who experienced primary and secondary recurrent pregnancy loss were included. PCR was performed in a 25 µL reaction solution containing template genomic DNA (10–50 ng), 40 µM each of dATP, dCTP, dGTP, dTTP (Biotools, B & M Labs, Madrid, Spain); 1 unit of *Taq DNA Polymerase* (Biotools, Madrid, Spain) with 10 µL of 10x *Taq DNA Polymerase* buffer (with 1.5 mM of MgCl_2_), and 1 µM primer in nuclease free de-ionized water as per the standardized protocol for better results (Lehmann et al., 2013).The PCR primer sequences for two SNPs are described in Supplementary Materials, Table S2. *BaeGI* (BioLabs, Bangalore, India) and *MspI* (Biolabs, Bangalore, India), restriction endonuclease enzymes were used to digest the 243 bp and 177 bp amplicon for 4 h and overnight at 37 °C respectively. The amplified product was subjected to electrophoresis for 1 h in a 3% agarose gel followed by ethidium bromide staining and ultraviolet illumination to identify the genotypes (Supplementary Materials, Figure S2a,b).To ensure the quality control, two independent observers randomly selected 5% samples from each group for genotyping to confirm the reproducibility of results.

### 2.6. High Resolution Melting (HRM) Analysis

**Figure 1:**
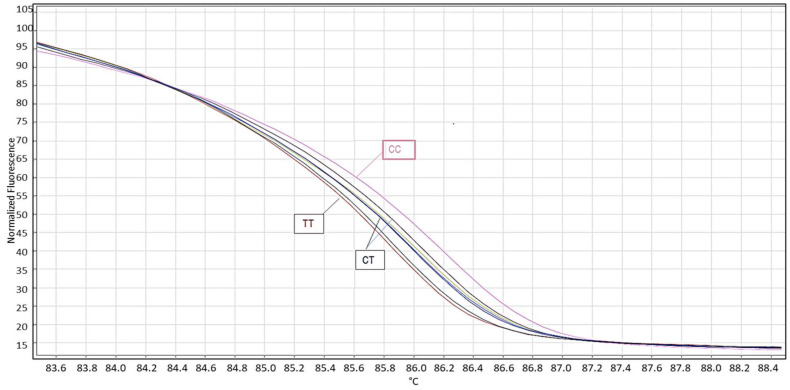
HRM analysis of miR-125a C/T polymorphism. Results were analyzed in the normalized fluorescence versus temperature plot, which indicates the normalized plot. Wild-type genotype are represented by TT while mutant genotypes represent CC and heterozygous, which contain both alleles, and are heteroduplex with different forms of CT.

The HRM method was also performed for the genotyping of miR-125ars12976445 C/T and rs10404453 A/G (QIAGEN Rotor-Gene® 6000) by using the following protocol: forward primer 0.2 µM and reverse primer 0.2 µM (primer sequences are listed in the Supplementary Materials, Table S2), ddH_2_O 5.8 μL, genomic DNA (10 ng/μL) 8–10 μL, HRM master mix KAPA HRM FAST Master Mix (2×) in a final volume of 20 μL. For miR-125a gene amplification, the thermocycler program was as follows: the initial PCR activation step (pre-amplification) at 95 °C for 5 min with a ramp rate of 4.4 °C/s; the amplification stage included 35 cycles of denaturation at 94 °C for 30 s with a ramp rate of 4.4 °C/s; annealing at 58 °C for 30 s with a ramp rate of 2.2 °C/s; and melting analysis at 72 °C for 30 min with a the ramp rate of 4.4 °C/s, at 70 °C for 1 s with the ramp rate of 4.4 °C/s, 90 °C continuous fluorescence data acquisition with the ramp rate of 0.02 °C/s and 25 acquisitions per second; then, the samples were cooled after HRM at 40 °C for 30 s with the ramp rate of 2.2 °C/s (Figure 1).

### 2.7. Sequencing

**Figure 2:**
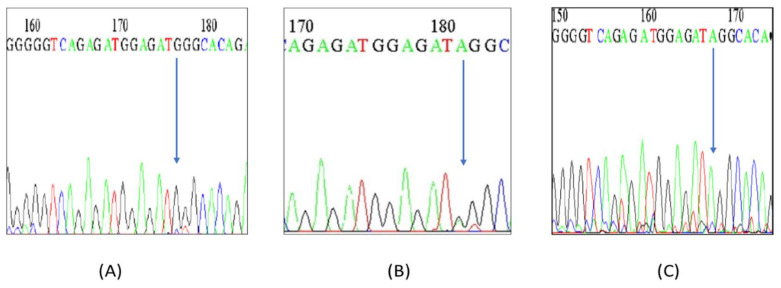
Reverse Sequencing of *miR*-125a C/T polymorphism showing partial electropherograms representing(**A**) variant CC, (**B**) heterozygous CT and (**C**) TT wild genotypes.

By using standard PCR protocols, DNA specimens were amplified (Lehmann et al., 2013), and subsequently, PCR products were sequenced in a reverse direction with the Sanger’s Sequencing platform. The sequencing results were analyzed using ABI Prism 3500, (Waltham, MA, USA) (Perkin Elmer) software as shown in Figure 2 with the help of Clustal Omega tool PCR primers used for miR-125a sequencing, as listed in the Supplementary Materials, Table S2.

### 2.8. Expression Analysis of miR-125a by q–PCR

For quantification of mature miR-125a, specific stem loop primers were used instead of oligo dT to generate specific cDNA of mature miR-125a by using miScript RT kit (Qiagen, Hilden, Germany). The sequence is listed in Supplementary Materials, Table S2. Expression was analyzed with the miScript SYBR Green PCR kit following the manufacturer’s manual. A QuantStudio™ 3 Real-Time PCR machine (Applied Biosystems, Waltham, MA, USA) was used to run the q–PCR and U6were used as an internal control to normalize the expression of miRNA. A 2^−ΔΔCT^ method was used to calculate the fold change (Rao et al., 2013). All the samples were taken in triplicate with three experimental replicates.

### 2.9. Ethical Compliance

All procedures performed in studies involving human participants were in accordance with the ethical standards of the institutional and/or national research committee and with the 1964 Helsinki declaration and its later amendments or comparable ethical standards, and ethical approval was obtained from Institutional Ethical Committee (SKIMS Study ref: Protocol 81/2014).

### 2.10. Statistical Analysis

IBM Statistics SPSS software (Version-23) and GraphPad Prism 8.0 (Graph-Pad Software, San Diego, CA, USA) were used to perform statistical analysis. The cases and controls were evaluated using the chi square test for categorical variables, such as sex and age, of the demographic variables. A goodness-of-fit chi-square test was performed to evaluate whether the polymorphisms were in Hardy–Weinberg equilibrium between cases and controls. Odds ratios (OR) were used as estimates of the relative risk, and 95% confidence intervals (CI) were calculated to estimate the association between certain genotypes or other related risk factors of RPL. Real-time data were expressed as Ct values and comparative analysis was performed using the Livac method (^ΔΔCt^). Results were presented as the fold change relative to the control group. All the data analyzed were indicated as the mean ± SD. The unpaired t-test was used for two group comparison and two-way analysis of variance (ANOVA) was used for analyzing the difference. A *p*-value < 0.05 was considered statistically significant. The sample size was verified using G*Power (V3.1.9.4) software.

## 3. Results

### 3.1. Genotyping of miR-125a by PCR-RFLP/HRM/DNA Sequencing

In this study, 150 patients with RPL and 180 healthy volunteers with atleast two full term pregnancies with no history of miscarriage were screened for polymorphic genetic analysis. Demographic and clinico-pathological characteristics such as age, number of miscarriages, consanguinity, family history and other related investigations such as TORCH, APLA, VDRL and USG findings were collected from the patients and are listed in the Supplementary Materials, Table S1. The demographic and clinico-pathological characteristics of the participants in the two groups did not differ significantly (*p* > 0.05) while consanguinity and family history showed differences when the RPL group was compared to the control group (*p* < 0.05).

Initially, two miR-125a SNPs were analyzed (rs12976445 and rs10404453) with the PCR-RFLP method. Three different genetic models were used to see the relation of different genotypes among cases and controls; the additive, dominant and recessive model (Table 1). 

When TT genotype was compared against CT + CC (dominant model), the difference in frequencies of genotypes among cases and controls was 34% (TT), 66% (CT + CC) and 41.1% (TT), 58.8% (CT + CC), respectively, with an OR =1.35 (CI = 0.86–2.1), showing a non- significant association (*p* > 0.05). In the case of the recessive model, no significant difference was seen in CC and CT + TT genotype among cases and controls (18.6% vs. 10.5% and 81.3% vs. 89.4%), respectively, with an OR = 0.7 (CI: 0.4–1.3) (*p* = 0.4). Interestingly, when TT was compared against CC, the variant genotype CC was found more frequently in RPL cases (35.4%) than controls (20.5%), with an OR = 2.2 (CI: 1.2–4.3), which showed a significant association (*p* = 0.02). The frequency of variant ‘C’ allele observed in RPL cases as well as controls was found to be 0.42 versus 0.34, respectively, with an OR =1.4 (CI: 1.0–1.90). This observation showed a significant association between variant allele in cases and controls (*p* = 0.04), as depicted in Table 1. Furthermore, 50 POC samples were analyzed for SNP rs12976445inmiR-125a gene and no significant differences were found in the frequency of variant genotypes among the two groups (*p* = 0.07) as shown in Table 2.

However, significant results were found for heterozygous CT genotype in POC cases and controls (*p* = 0.02). Besides, the same series of POC samples were evaluated for another rs10404453, but the comparison between the two groups showed insignificant differences. Thus, in all cases (RPL cases, POC samples and controls), this SNP is not informative (monomorphic).

After stratification, with respect to different risk parameters, RPL cases who had experienced <3 miscarriages were significantly associated with combined variant genotype (CT + CC) of rs12976445 C/T. Here, the frequency was 73% in RPL cases against 59% in full-term healthy controls with an OR of 1.9 (CI: 1.04–3.49) (*p* = 0.04). All other clinical parameters such as the age, consanguinity and family history of the RPL cases did not associate with rs12976445 C/T, as shown in Table 3.

Furthermore, we used the HRM analysis, which was performed on 20 randomly selected samples where the curves were analyzed using QIAGEN Rotor-Gene® 6000 software version 1.7.87. The main purpose of this technique was to develop a sensitive test that can rapidly detects SNPs in various genes. It is worth mentioning that our results from the PCR-RFLP were consistent with that of the HRM. Figure 1 represents themiR-125a SNP rs12976445 according to HRM.

Direct sequencing for the SNP rs12976445inmiR-125a genes in a randomly selected group of 20 enrolled patients with RPL was also performed and we found our results were concordant with that of the PCR-RFLP method, revealing a significant difference in genotype frequency between control subjects and patients with RPL. Partial electropherograms of the miR-125a C/T polymorphism showing (A) variant CC, (B) heterozygous CT and (C) TT wild genotypes are presented in Figure 2.

### 3.2. Expression Analysis of miR-125a with Respect to pri-miR-125a SNP

**Figure 3:**
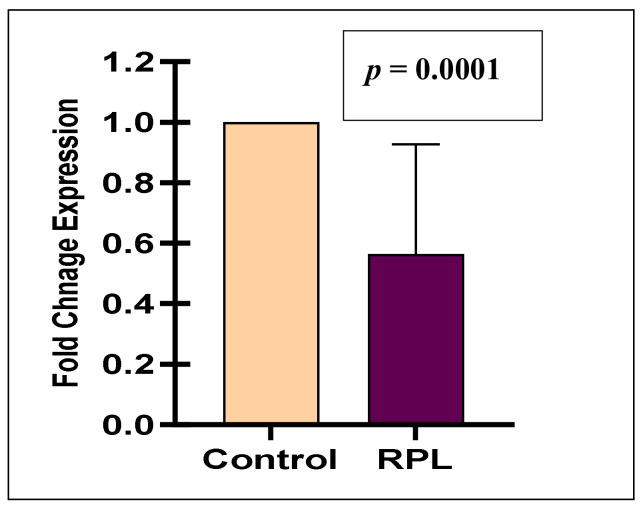
Relative expression of *miR*-125a in study subjects.

To investigate the expression of miR-125ain RPL patients, the expression levels of miR-125a was confirmed by q-PCR analysis, which revealed the expression of miR-125a was significantly lower in the plasma of the RPL group compared to the control group (*p* = 0.0001) (Figure 3).

**Figure 4:**
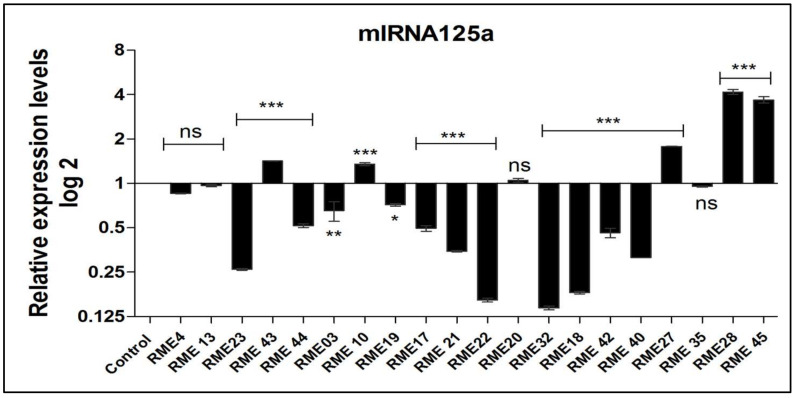
Representative figure for the relative expression analysis of miR-125a (*** cases represents highly significant, ** significant and * less significant. ns represents non- significant).

In 40 RPL cases, variations in the expression of miR-125a (low and high expression levels) were observed whereby the mean level of 62% of RPL cases showed a 2-fold decrease in expression and 38% of the cases showed a 2.24-fold increase in expression. Based on log2 relative expression, of the samples that demonstrated low expression levels (62%),45% were highly significant *** (*p* = 0.001),5%were significant ** (*p* < 0.05) and 5% showed less significance * (*p* < 0.05) while the rest of the (15%) samples were non-significant (*p* > 0.05). Only 38% (*n* = 15) of the RPL cases were identified as up-regulated formiR-125a expression wherein 25% were highly significant and the rest showed no significant pattern (*p* > 0.05) as depicted in Figure 4.

**Figure 5:**
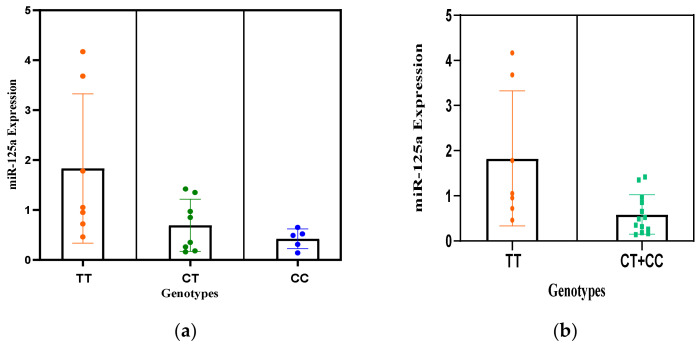
Scatter plot for miR-125a expression levels in association with the genotypes of miR-125a rs12976445 C/T polymorphism. (**a**) miR-125a expression levels with each genotype of miR-125a rs12976445 where TT represents wild genotype, CT heterozygous and CC homozygous mutant. (**b**) miR-125a expression levels with respect to TT vs. combined genotype CT+ CC.

Polymorphic sequence variations in the same group of participants were analyzed for miR-125a SNPs (rs12976445C/T). Genotypes from the data for rs12976445 C/T polymorphism showed significance among patients with miR-125a expression. A scatter plot illustrates the miR-125a expression levels and genotypes of miR-125a (Figure 5a,b).

**Figure 6:**
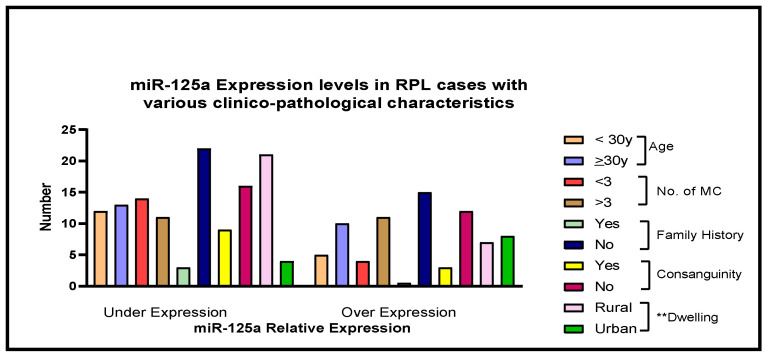
The association between different clinico-pathological variables and miR-125a expression levels in Recurrent Pregnancy Loss (RPL) cases (** represents significant, MC; miscarriage).

Significantly low expression of miR-125a was observed with respect to rs12976445 C/T (*p* < 0.05) (Table 4) wherein patients with heterozygous CT and homozygous CC genotype for the miR-125a showed significantly decreased expression of miR-125a, that is, 2.17-fold (*p* = 0.03) and 2.3-fold (*p* = 0.02), respectively. Further, different clinico-pathological parameters and miR-125a expression in RPL cases showed no statistical significance, except the dwelling of the patients (*p* = 0.02) (Figure 6).

### 3.3. Multivariat Analysis

Multivariate analysis was performed to determine the influence of various demographic features, rs12976445 genotypes and miR-125a expression on the occurrence of RPL and showed that none of the demographic features were related. However, for rs12976445 SNP, the presence of CC genotype was found to confer a nearly 2-fold increased risk of pregnancy failure (HR = 1.786, 95%CI = 0.39–4.61, *p* = 0.037). Similar results were observed for mir-125a whereby its decreased expression increased the risk of the RPL by nearly 3-fold (HR = 2.786, 95%CI = 0.84–5.33, *p* = 0.008) after adjusting for other test variables such as age, family history, consanguinity (Supplementary Materials, Table S3).

## 4. Discussion

A number of studies have shown that the regulatory capacity of miRNAs is affected by polymorphism, thereby influencing miRNA processing and/or interactions. In our study, we hypothesized that miR-125a plays an influential role in the regulation of genes involved in a successful pregnancy. Thus, we presented a case-control study of two polymorphisms from miR-125a (rs 12976445 C/T and rs 10404453 A/G) to analyze its impact on miR-125a expression in recurrent pregnancy loss cases. In this study, the miR-125a genotyping was not only done by RFLP but was evaluated for validation by HRM analysis. The results obtained from both assays were finally confirmed by gold standard DNA sequencing method to confirm the status of different genotypes. It is a standard DNA sequencing method to confirm the status of different genotypes and it is already known through various earlier studies that compared various HRM models to clearly demonstrate sequence variants [30,31,32]. To measure the impact of variant genotypes, an evaluation of miR-125a expression was done in RPL patients in comparison to women with normal full-term pregnancies as the control population. A study reviewed the performance of different methods for BRCA1/2 and demonstrated the need for enhanced statistical significance of studies that could thoroughly analyze the screening efficiency of newer sequence variants in order to develop more confidence in the findings [33]. Our study included 330 samples from RPL cases and healthy controls, where the latter included subjects of proven fertility with normal menstrual cycles without a history of pregnancy failure. Samples were taken from controls at the gestational age corresponding to each of the events in RPL cases. Exactly 20 randomly selected samples were screened for HRM analysis. The study found that HRM analysis is a highly sensitive technique for detecting all forms of sequence variants such as heterozygous and homozygous conditions with an overall sensitivity of 100%.

In this study, miR-125a rs12976445 C/T polymorphic variation revealed that homozygous CC variant genotype and combined variant (CT + TT) were associated with risk of RPL. However, the genotypes of rs10404453 polymorphism existed as wild type monomorphic only. Our study agrees with an earlier functional investigation conducted by Yi et al., 2011 who furnished the first evidence that supports the connection between the functional polymorphisms in pri-miR-125a and susceptibility to RPL [10]. The miR-125a (rs 12976445) results from our report match those of Hu et al. where variant genotype/allele (CC, C) showed an association with RPL cases. There are reports on miRNAs that contain many polymorphic sequence variations and they have been identified as having a plausible link with various diseases [34,35,36,37]. Moreover, another study found that miR-125a rs12976445 C/T polymorphism is linked with the pathogenesis and prognosis of autoimmune diseases [27]. Our results corroborate those of Inoue et al. which showed variant C allele and CC genotype are associated with Hashimoto and Graves’ disease intractability. Another earlier report showed that C allele carriers (CC + CT genotypes), which were also associated with RPL cases in our report, showed a connection with low miR-125a expression as against the TT genotype [38]. It is suggested that miR-125a rs12976445 C/T may be associated with the development of multiple diseases due to its plausible link to pri-miR processing, rather than owing to the varied expression levels of miR-125a [26]. In our study, multivariate analysis showed the influence of CC genotype and low expression of miR-125a on the risk of RPL and a similar scenario has been observed in gastric cancer patients where low expression of miR-125a is associated with the overall survival of patients [39]. The monomorphic nature of miR-125a (rs10404453) SNP is not only reported in this study, but has been found in an earlier study done on autoimmune disorders in the Japanese population [27]. The importance of miR-125a SNPs with regard to RPL is further validated by a functional analysis that demonstrated that pri-miR-125a mutant genotype can increase the endometrial stromal cell invasive effectiveness and enhance the sensitivity of cells to mifepristone-induced inhibition of cell proliferation as against the wild type genotype. The finding suggests that A > G mutations in the pri-miR-125a coding region coalesce with the minor variant alleles of rs41275794 and rs12976445, which poses a genetic risk to RPL by disturbing the expression of miR-125a. This subsequently interferes with the regulation of the gene network between miR-125a and mRNA [28]. The study also showed a significant association of heterozygote CT and allele C of miR-125a in the product of conception and controls. This shows the plausible impact of variant allele on the outcome of RPL cases in the current study. Since no previous study seems to have studied the POC with reference to miRNA sequence variations, the discussion of its implication with respect to other studies cannot be done. Moreover, different clinical factors did not show any association with respect to rs12976445 C/T except the patients that experienced <3 miscarriages showed an association with the variant combined genotype.

To further discern its biological role, miRNA expression was investigated in the current study and this identified a significant association with RPL. Our study found that miR-125a showed moderately decreased expression in RPL cases compared to controls. In the series of RPL cases, different patterns of expression were detected. wherein under expression and over expression of miR-125a wase found in 25 (62%) and 15 (38%), respectively, plasma samples from RPL patients. In contrast, the study conducted by Hosseini et al. on early pregnancy losses in the Turkish population demonstrated that the expression of miR 125a 3p, (a mature type of miR) was elevated in villous tissue samples from RPL cases [40]. Along the same lines, Li and Li (2016) confirmed that miR-125a expression showed an elevation in idiopathic recurrent spontaneous abortion samples when compared against control samples, and another investigation demonstrated the plausible role of miR-125a expression and its association with RPL [10,41]. Deregulation of miRNA has been connected with many diseases and among them, many studies have observed a plausible link to different cancers [18,42,43], but the precise function and fundamental molecular insights into miRNAs continue to be delineated. As decreased expression of miR-125a was related to RPL cases in our study, a similar scenario for this miRNA has been seen in various cancers that implicate its role in diseases unrelated to RPL; these predominantly include hepatocellular cancer [44], colorectal cancer [45], esophageal and stomach cancer [46,47]. Low expression of miR-125a has been observed to be associated with poor prognosis in CRC [45], HCC [44] and gastric cancer [47]. This evidence substantiates that miR-125a playsthe role of a tumor suppressor gene. A study shows that miR-125a was seen to differentially express in the implantation versus the pre-implantation of rat uteri. This observable fact suggestsa rational likelihood that atypical expression of miR-125a contributes to the implantation malfunction that is one of major causes of RPL [48,49]. It is now believed that miR-125a is an example of a miRNA with a range of diverse functions in a spectrum of tissues. As discussed, miR-125a is accepted as a vital constituent of the gene regulatory system and its deregulation in expression in different human diseases and our study, in conformation with other referred studies, pointsto its major role in recurrent pregnancy losses. 

Furthermore, the risk factors associated with RPL patients such as age, consanguinity and multi-generation family cases in our study werenot associated with expression of miR-125a, except in patients from different locations. Since no single international study has conducted any such analysis, these parameters could not be discussed with reference tothe global context.

Moreover, further studies on the functional role of miR-125a will help in understanding the progression and pathogenesis of RPL.

## 5. Conclusions

It is concluded thatmiR-125a rs12976445C/T increases the risk for a genetic predisposition to suffer RPL in our population. Our study indicates that rs12976445 polymorphism may be related to RPL through a potential function of pri-miRNA processing. The alteration of miR-125a subsequently causes decreased miR-125a expression in RPL cases. Significantly higher expression of miR-125a with respect to rs12976445 TT genotype shows that miR-125a is a vital constituent of the gene regulatory system and its deregulation in expression contributes to RPL pathogenesis. Since only a few reports are available, there is a need to investigate the genetic SNPs of miR-125a in a large sample of RPL cases.

## Figures and Tables

**Table 1:** Single nucleotide polymorphism (SNP) genotype distribution of miR-125a (rs12976445 C/T and rs10404453 A/G) and allele frequencies in RPL cases and control women.

SNP Single Nucleotide Polymorphism	Genotype	Case *n* = 150 (%)	Control *n* = 180 (%)	OR (95CI%)	*p* Value
rs12976445 C/T Overall Genotyping	TT CT CC	51 (34) 70 (46.6) 29 (19.3)	74 (41.1) 87 (48.3) 19(10.5)	Ref 1.1 (0.7–1.9) 2.2 (1.2–4.3)	Ref 0.5 0.02
Dominant Model	TT CT + CC	51 (34) 99 (66)	74 (41.1) 106 (58.8)	Ref 1.35 (0.8–2.1)	Ref 0.21
Recessive Model	CC CT + TT	29 (18.6) 121 (81.3)	19 (10.5) 106 (89.4)	Ref 0.7 (0.4–1.4)	Ref 0.4
Additive Model	TT CC	51 (64.5) 29 (35.4)	74 (79.5) 19 (20.5)	Ref 2.2(1.2–4.3)	Ref 0.02
Allele Frequency	T C	172 (57.6) 128 (42.3)	235 (65.2) 125 (34.7)	Ref 1.4 (1.0–1.9)	Ref 0.04
rs10404453 A/G Overall Genotyping	GG AG AA	150 (100) 0 0	180 (100) 0 0		Ref 0.70 0.70

OR, odds ratio; OR 95 CI%, 95% confidence interval; Ref, reference.

**Table 2:** Single nucleotide polymorphism (SNP) genotype distribution and allele frequencies of *miR*-125a (rs 12976445 C/T) in POC samples in contrast to controls.

SNP	Genotype	POC Product of Conception *n* = 50(%)	Control *n* = 180 (%)	OR (95 CI%)	*p* Value
rs12976445 C/T Overall Genotyping	TT CT CC	11 (22) 31 (62) 8 (16)	74 (41.1) 87 (48.3) 19 (10.5)	Ref 2.4 (1.1–5.09) 2.8 (1.0–8.0)	Ref 0.02 0.07
Recessive model	CC CT + TT	08 (16) 42 (84)	19 (10.5) 161 (89.4)	Ref 0.9 (0.3–2.7)	Ref 0.9
Allele Frequency	T C	53 (53) 47 (47)	235 (65.2) 25 (34.7)	Ref 1.7 (1.06–2.6)	Ref 0.02

OR, odds ratio; OR 95 CI%, 95% confidence interval; Ref, reference.

**Table 3:** Genotypic distribution of SNP rs12976445 C/T in RPL cases and healthy controls with different clinico-pathological characteristics.

SNP	Case (%)		Control (%)		OR (95 CI %)	*p* Value
	TT	CT + CC	TT	CT + CC		
Age <30 ≥30	21 (27) 30 (41)	56 (73) 43 (59)	32 (40) 42 (42)	48 (60) 58 (58)	1.79 (0.9–3.4) 1.03 (0.56–1.91)	0.1 0.9
Miscarriages <3 ≥3	19 (27) 32 (41)	52 (73) 47 (59)				0.04 * 0.09
Family History Yes No	10 (43) 41 (32)	13 (57) 86 (68)	4 (40) 70 (41)	6 (60) 100 (59)	0.8 (0.1–3.9) 1.4 (0.9–2.3)	0.9 0.1
Consanguinity Yes No	09 (28) 42 (36)	23 (72) 76 (64)	8 (29) 66 (43)	20 (71) 86 (57)	1.0 (0.3–3.1) 1.3 (0.8–2.2)	0.9 0.2

OR, odds ratio; OR 95 CI%, 95% confidence interval (* cases were compared with respect to the overall frequency of controls); Ref, reference; TT (wild) vs. CT (heterozygous) + CC (Homozygous mutant).

**Table 4:** miR-125a expression in plasma samples of RPL cases in relation to genotypes.

Expression Analysis of miR-125a in Plasma Samples of RPL Cases				
Genotypes	*n* = 40(%)	Mean ± SD Fold Change		*p* Value
		Under	Over	
rs12976445 C/T				
TT	13(32.5)	4(31)0.71 ± 0.24	9(69)2.67 ± 1.49	Ref
CT	22(55)	16(73)0.46 ± 0.35	6(27)1.38 ± 0.04	0.03
CC	5(12.5)	5(100)0.42 ± 0.19	0	0.02
CC + CT	27(67.5)	21(77.7)0.44 ± 0.28	6(22.2)1.38 ± 0.04	0.006

## Supplementary Materials

The following supporting information can be downloaded at: https://www.mdpi.com/article/10.3390/jcm11133834/s1, Figure S1: Path map showing the selection of patient with recurrent pregnancy loss and control group; Table S1: Demographic features of the subjects enrolled in the study (Recurrent Pregnancy Losses). Table S2: The primers, polymerase chain reaction (PCR) conditions and a restriction enzyme used in this study. Table S3: Multivariat analysis of demographic characteristics, rs 12976445 genotypes and miR-125a expression in Recurrent Miscarriage patients. Figure S2: RFLP picture of miR-125a rs 12976445 C/T after restriction digestion with BaeGI, (3%) agarose gel electrophoresis, Lane M: 100 bp marker, Lane 2,4: homozygous variant CC genotype, Lane 1,3,5: homozygous wild TT genotype, Lane 6,7: heterozygous CT genotype. Figure S3: RFLP picture of miR-125a10404453 A/G after restriction digestion with MspI, (3%) agarose gel electrophoresis, Lane M: 50 bp marker, Lane1-7: homozygous CC genotype.
