# Supplementary material for: Implications of Decreased Expression of miR-125a with Respect to Its Variant Allele in the Pathogenesis of Recurrent Pregnancy Loss: A Study in a High Incidence Zone
Source: J Clin Med. 2022 Jul 1;11(13):3834. doi: 10.3390/jcm11133834 (PMC9267497; doi:10.3390/jcm11133834)
Supplement: Supplementary file 1 [file jcm-11-03834-s001.zip › jcm-1721312-supplementary.pdf]

Table S1: Demographic features of the subjects enrolled in the study (Recurrent Pregnancy Losses)

**Demographic Features**	**Cases**<br>**n=200 (%)**	**Controls**<br>**n= 240 (%)**	***p* value**
**Age**			
< 30	112 (56)	125 (52.1)	Reference (Ref)
≥ 30	88 (44)	115 (47.9)	0.25
**Miscarriages**			
<3	79 (39.5)		
≥ 3	121 (60.5)	-	
**Consanguinity**			
Yes	70 (35)	41 (17)	Ref
No	130 (65)	199 (83)	**<0.05**
**Family History**			
Yes	39 (19.5)	13 (5.4)	Ref
No	161 (80.5)	227 (94.6)	**<0.05**
**TORCH**			
Positive	4 (2.6)	0	
Negative	148 (97.3)	240 (100)	
**APLA**			
Positive	2 (2.5)	0	
Negative	77 (97.5)	240 (100)	
**VDRL**			
Positive	0	0	
Negative	152 (100)	240 (100)	
**USG Findings**			
Normal	101(78.3)	188 (98.4)	
Abnormal	28 (21.7)	3 (1.57)	

Ref, Reference

Table S2: The primers, polymerase chain reaction (PCR) conditions and a restriction enzyme used in this study

**Primer Pairs**		**PCR conditions**	**Restriction Enzymes**
**PCR–RFLP rs12976445 C/T**	5′-TTTTGGTCTTTCTGTCTCTGG -3′<br>5′-TGGAGGAAGGGTATGAGGAGT-3′	95°C for 5 min (94°C for 30 s, 58°C for 30 s, 72°C for 30 s) × 35 cycles72°C for 7 min	**BaeGI**
**Sequencing rs12976445 C/T**	5′-TTTTGGTCTTTCTGTCTCTGG -3′<br>5′-TGGAGGAAGGGTATGAGGAGT-3′	95°C for 5 min (94°C for 30 s, 58°C for 30 s, 72°C for 30 s) × 35 cycles72°C for 7 min	**-**
**PCR–RFLP rs10404453 A/G**	5′-CTGACTCCCTCTTATTCTGG-3′<br>5′-TAGAGACTGGCAACATGG-3′	95°C for 5 min (94°C for 30 s, 55°C for 30 s, 72°C for 30 s) × 35 cycles72°C for 7 min	**MspI**
**miR-125a**	F-5'GGTGTCCCTGAGACCCTTTAA-3'<br>R-5' GTGCAGGGTCCGAGGT		

RNU6	F-5' GCTTCGGCAGCACATATACTAAAAT<br>R-5'AACGCTTCACGAATTTGCGT

Table S3: Multivariate analysis of demographic characteristics, rs 12976445 genotypes and miR-125a expression in Recurrent Miscarriage patients

Parameter	Hazard (B)	95% CI	*p* Value
**Age**	1.260	0.78- 2.13	0.308
**Family History**	1.471	0.80- 3.20	0.183
**Consanguinity**	0.198	0.46-1.45	0.500
**rs12976445 CT**	1.266	0.14- 3.99	0.197
**rs12976445 CC**	1.788	0.39- 4.61	0.037
**miR-125a**	2.786	0.84- 5.33	0.008

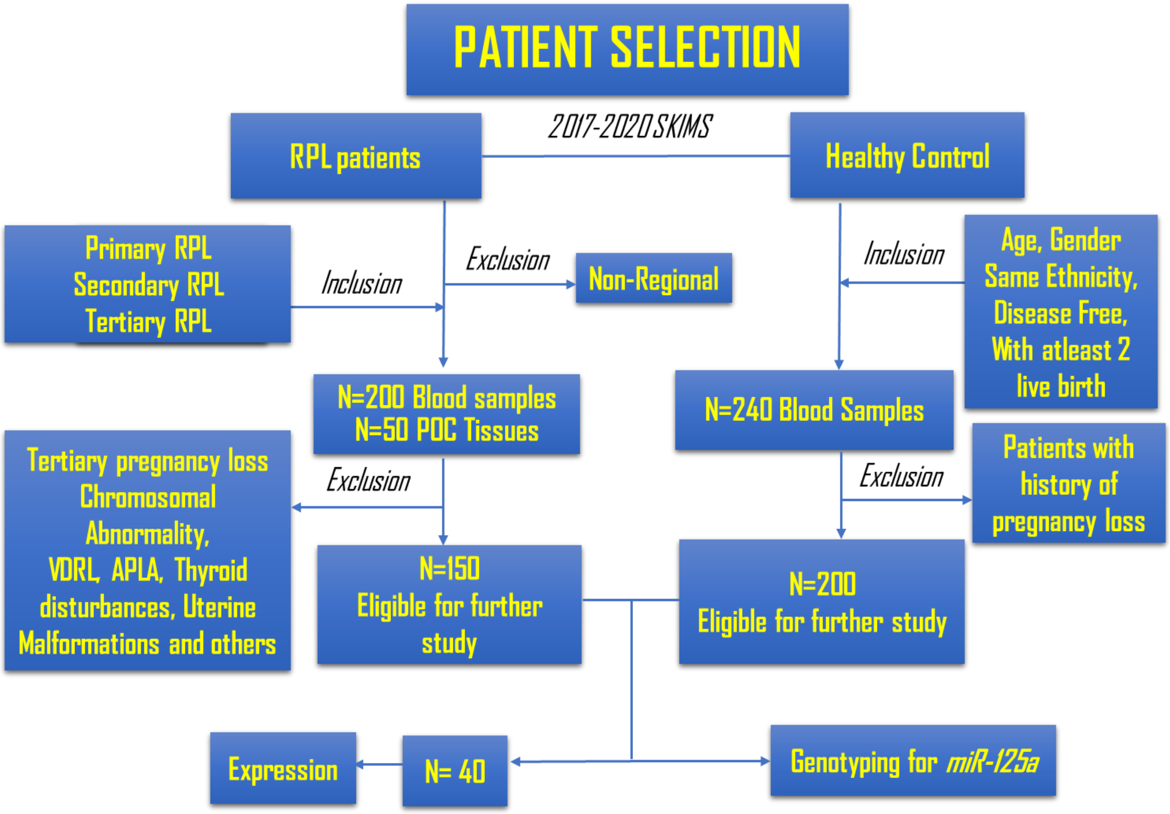

Figure S1: Path map showing the selection of patient with recurrent pregnancy loss and control group.

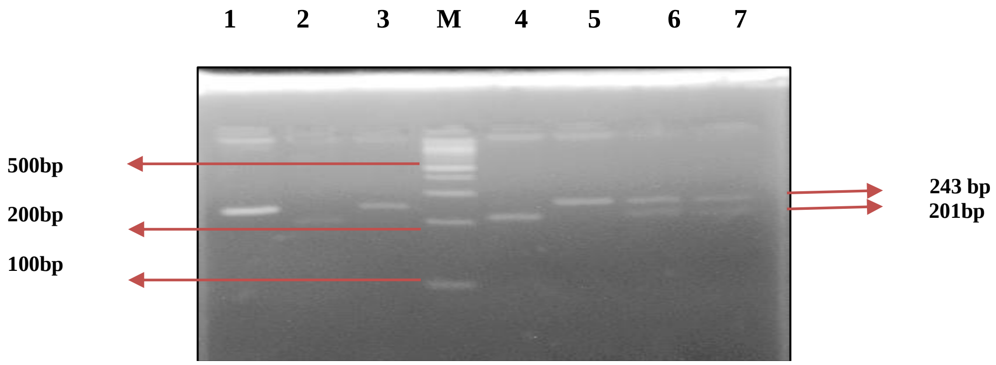

Figure S2 (a): RFLP picture of *miR-125a rs* 12976445 C/T after restriction digestion with *BaeGI*
(3%) agarose gel electrophoresis
Lane M: 100bp marker, Lane 2,4 : homozygous variant CC genotype
Lane 1,3,5: homozygous wild TT genotype
Lane 6,7 : heterozygous CT genotype

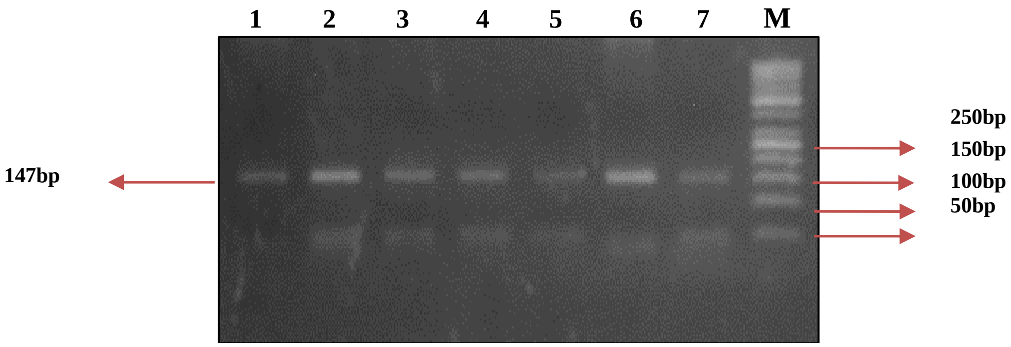

Figure S2 (b): RFLP picture of *miR-125a* 10404453 A/G after restriction digestion with *MspI*
(3%) agarose gel electrophoresis
Lane M: 50bp marker, Lane1-7 : homozygous CC genotype
